# Data on household energy consumption in small urban & rural settlements of Georgia

**DOI:** 10.1016/j.dib.2019.103859

**Published:** 2019-03-20

**Authors:** Giorgi Lekveishvili

**Affiliations:** Ivane Javakishvili Tbilisi State University, Georgia

**Keywords:** Household, Energy consumption, Heating, Georgia, Caucasus

## Abstract

The data is based on 303 interviews of households residing in stand-alone single-family (detached) buildings in small urban and rural settlements outside the large urban agglomerations in Georgia. The original data included household size (number of household members), total and heated areas of building owned by the household, heating source, total annual energy consumption and expenditures for each household, as well as energy consumption and expenditures by types of energy sources. The data fully reflects the behavioral differences and similarities between different types of households.

**Table:** Specifications table

Subject area	*Energy geography, social economics*
More specific subject area	*Population energy consumption*
Type of data	*Table, figure, MS Excel file*
How data was acquired	*Survey*
Data format	*Raw & analyzed*
Experimental factors	*The total number of selected households were 303 in small urban and rural settlements in Georgia*
Experimental features	*Survey was carried out in households using a questionnaire*
Data source location	*Small urban and rural settlements of Georgia (4 watersheds, 8 sub-watersheds, 13 municipalities)*
Data accessibility	*Data is within this article*
Related research article	*Lekveishvili G., 2015, Spatial Analysis of Energy Consumption Structure of Residential Sector (Household) in small Urban and Rural Settlements of Georgia. Journal of Young Researchers (jyr.tsu.ge). Ivane Javakhishvili Tbilisi State University.*

**Table:** 

**Value of the data** • The data can be used to establish correlations between household energy consumption/expenditures and geography, demography, type of energy sources used, household building size and size of heated part of the building. • The data contains unique information on energy consumption as well as on energy expenditures in the residential sector of Georgia by types of energy sources. • The data can be used as an example to analyze household energy consumption for other regions with similar patterns of the household's energy use (Caucasus, parts of Eastern Europe, Post-Soviet/Post-communist states and etc.). • The data provides characteristics of typical household building size and heated area across the Georgia.

## 1 Data

The presented data is given in the form of tables. Its contents include type of settlement, number of household members (NHM), energy source used for heating (Heat_S), total building area (Total Area), heated area (H_Area), total annual energy consumption (Total_KWH), expenditures (Total_GEL), consumption and expenditures for each type of energy source – electricity (EL_KWH & EL_GEL), natural gas (NG_KWH & NG_GEL), liquid petroleum gas (LPG_KWH & LPG_GEL), firewood (Wood_KWH & Wood_GEL), other types of energy sources (Other_KWH & Other_GEL). Energy consumption is presented in kWh for each type of energy source and expenditures are presented in the Georgian national currency Lari (GEL). Using this data, we can count and analyze relations between the variables.

## 2 Experimental design, materials, and methods

### 2.1 Surveyed area

The survey was conducted in four watersheds (9 sub-watersheds) of Georgia and covered 13 municipalities across the country.

### 2.2 Questionnaire

The survey questionnaire was structured as follows:

1. The basic information about the household –settlement type, number of household members;
2. Basic information about energy consumption – total household energy consumption, and by energy sources;
3. Information about residential building – construction and reconstruction dates of the building, total and heated areas.
4. Energy expenditures – household annual expenditures by types of energy.

### 2.3 Annual household energy consumption

**Fig. 1:**
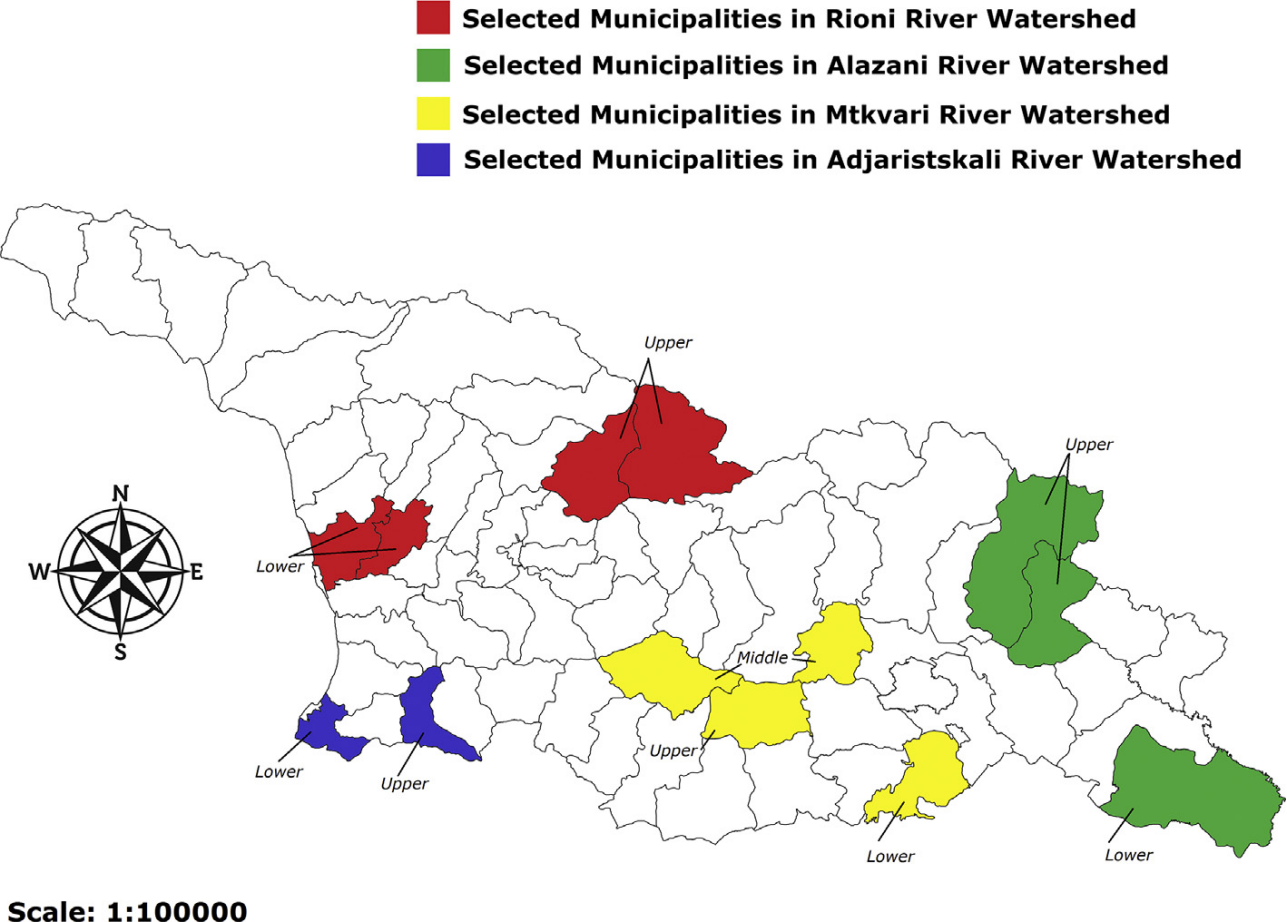
Selected watersheds and municipalities.

Typical household buildings are quite old (on average 40–50 years old). Houses that were built after the early 1980s are scarce. Most of the old household buildings have never been renovated. Vast majority of houses are characterized by inadequate thermal properties of building envelope resulting in very high energy losses (see Fig. 1).

**Fig. 2:**
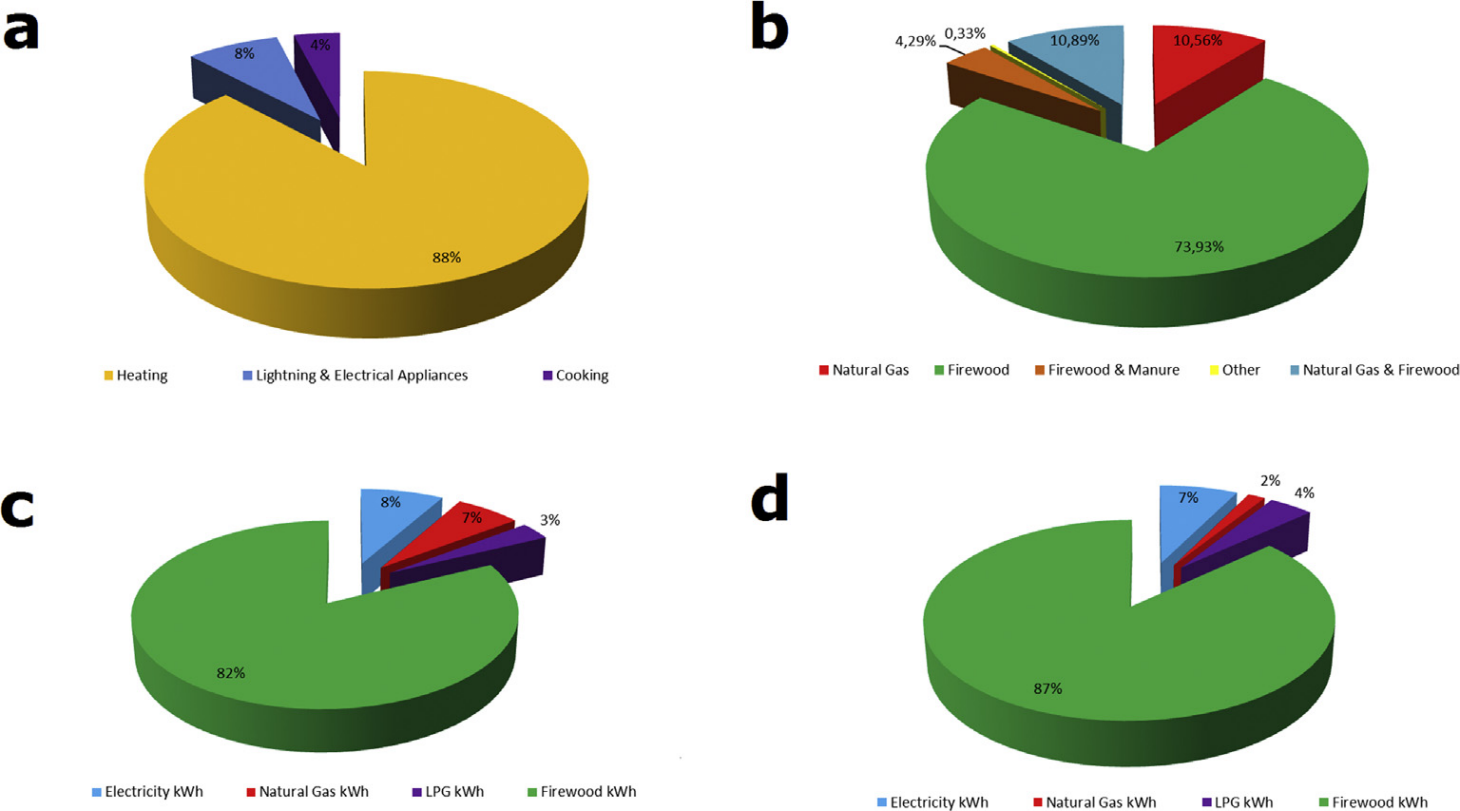
(a) Average household energy consumption structure by types of end use % (b) Heating sources of surveyed households % (c) Average urban household energy consumption structure by types of energy % (d) Average rural household energy consumption structure by types of energy %.

Households mainly consume energy for heating purposes (Fig. 2 a). The main heating source for population of these settlements is firewood (Fig. 2 b) burnt in wood stoves that are of the old type dating back to the beginning of 20th century (or even earlier).

Therefore calculation was carried out for average annual energy consumption structure for urban and rural households that use firewood as a heating source (Table 1, Fig. 2 c and d).

**Table 1:** The average energy consumption structure in small urban and rural settlements,kWh.

Sub-Watershed		Electricity kWh	Natural Gas kWh	LPG kWh	Firewood kWh	Total kWh
Lower Adjaristskali	Urban	2260	0	1467	11273	15000
	Rural	2003	0	1500	13127	16632
Lower Alazani	Urban	1083	2442	92	14596	18214
	Rural	740	1872	112	23949	26673
Lower Mtkvari	Urban	1080	6739	0	10680	18499
	Rural	1110	3931	0	8900	13941
Lower Rioni	Urban	2313	0	1510	29310	33134
	Rural	1783	0	1133	24518	27435
Middle Mtkvari	Urban	1548	4492	0	8306	14347
	Rural	1906	187	962	13528	16584
Upper Adjaristskali	Urban	1920	0	1042	15575	18537
	Rural	1280	0	1171	12905	15356
Upper Alazani	Urban	1641	1661	231	14240	17774
	Rural	1405	541	733	18302	20981
Upper Mtkvari	Urban	2000	0	1184	20173	23357
	Rural	1383	0	1263	15210	17858
Upper Rioni	Urban	1660	1010	186	17622	20479
	Rural	1762	0	284	21995	24042
Average	Urban	1740	1376	663	17434	21214
	Rural	1565	346	896	18530	21339

For more information on survey see [1].

## References

[1] Lekveishvili G. (2015). Spatial Analysis of Energy Consumption Structure of Residential Sector (Household) in Small Urban and Rural Settlements of Georgia. Journal of Young Researchers.

