# Supplementary material for: Data on household energy consumption in small urban & rural settlements of Georgia
Source: Data Brief. 2019 Mar 20;24:103859. doi: 10.1016/j.dib.2019.103859 (PMC6480931; doi:10.1016/j.dib.2019.103859)
Supplement: Supplementary file 1 — Multimedia component 1 [file mmc1.doc]

**DECLARATION OF CONFLICT OF INTEREST**

28 January, 2019

Tbilisi, Georgia

To the Data in Brief editor

Dear Sir/Madam,

I, Giorgi Lekveishvili write to declare that there is no conflict of interest to my data paper “**Data on Household Energy Consumption in Small Urban & Rural Settlements of Georgia”**


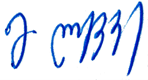
Sincerely Yours,

Giorgi Lekveishvili

+995598501679
